# Temporal Trends of Mitral Valve Prolapse-Attributable Mortality in Europe, 2012-2021

**DOI:** 10.1016/j.jacadv.2025.102350

**Published:** 2025-11-13

**Authors:** Marco Zuin, Matteo Bertini, Victoria Delgado, Patrizio Lancellotti, Claudio Bilato, Gianluca Rigatelli, Giuseppe Boriani, Gregory Piazza

**Affiliations:** a Department of Translational Medicine, University of Ferrara, Ferrara, Italy; b Department of Cardio-Thoraco-Vascular Sciences and Public Health, University of Padova, Padua, Italy; c Division of Cardiology, South Padova General Hospitals, Monselice, Italy; d Cardiology Unit, Department of Translational Medicine University of Ferrara, Azienda Ospedaliero-Universitaria S.Anna, Ferrara, Italy; e Department of Cardiology, Hospital University Germans Trias I Pujol, Badalona, Spain; f Department of Cardiology, University Hospital of Liège, GIGA Institutes Cardiovascular Sciences & Metabolism, Liège, Belgium; g Division of Cardiology, West Vicenza General Hospital, Arzignano, Italy; h Cardiology Division, Department of Biomedical, Metabolic and Neural Sciences, University of Modena and Reggio Emilia, Policlinico di Modena, Modena, Italy; i Thrombosis Research Group, Harvard Medical School, Brigham and Women's Hospital, Boston, Massachusetts, USA; j Division of Cardiovascular Medicine, Brigham and Women's Hospital, Boston, Massachusetts, USA

**Keywords:** mitral valve prolapse, mortality, trend

**Figure:**
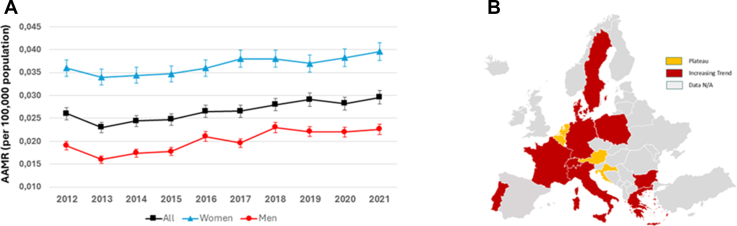

**What is the clinical question being addressed?**

Is mortality attributable to MVP changing over time in Europe, and what are the demographic and geographic patterns associated with these trends?

**What is the main finding?**

MVP-attributable mortality in Europe increased significantly from 2012 to 2021, particularly among older adults, women, and in specific countries like Italy, with no observed declines in any of the studied nations.

Mitral valve prolapse (MVP) is a common heart valve disorder affecting over 170 million people, with a rare malignant form linked to arrhythmias and sudden cardiac death (SCD).^1^ Challenges in assessing MVP-related mortality stem from inconsistent definitions, limited autopsy data, and nonstandardized reporting.^2^

## Objective

We analyzed MVP-attributed mortality trends in Europe using World Health Organization (WHO) data from 2012 to 2021, aiming to describe its epidemiology and assess its impact on overall mortality.

## Methods and findings

Data on MVP-attributable mortality were extracted from the publicly available WHO mortality data set,^3^ using the International Classification of Diseases-10th Revision (ICD-10) code I34.1 listed as the primary cause of death on the death certificate across European countries, covering the period from January 2012 to December 2021. By specifically selecting ICD-10 code I34.1, other mitral valve disease causes potentially leading to death were excluded. Attribution of MVP as the cause of death was based on physician certification and subsequent national coding processes. This study was conducted in accordance with the Strengthening the Reporting of Observational Studies in Epidemiology guidelines, and the methodological approach is consistent with a previous epidemiological investigation that evaluated trends in MVP-attributable mortality rates using ICD-10 codes.^4^ The study did not require Institutional Review Board approval since the analysis was based on deidentified and publicly available data.

Only European countries reporting MVP-attributable deaths were included into the analysis: Austria, Belgium, Bulgaria, Croatia, Denmark, France, Germany, Italy, the Netherlands, Poland, Portugal, Sweden, Spain, and Switzerland. Population estimates and mortality trends were stratified by age and sex. Age-adjusted mortality rates (AAMRs) per 100,000 individuals and their 95% CIs were calculated using standardization based on the 2013 European Standard Population. Joinpoint regression analysis was used to assess the trends in MVP-attributable mortality. European and Nationwide annual trends were assessed through average percent change (APC) and average annual percent change (AAPC) with relative 95% CIs. The number and location of joinpoints were determined using the default permutation test method (Monte Carlo, overall significance level = 0.05) testing models with 0 to 3 joinpoints and selecting the most parsimonious model based on the Bayesian Information Criterion. If no joinpoints were identified, a simple linear regression model was additional fitted to confirm trend estimates. Autocorrelation of residuals was assessed using the Durbin–Watson test. Paired comparison tests (p for parallelism) for subgroup trends were performed. Statistical analyses were performed using the Joinpoint regression (Joinpoint, version 4.6.0.0, National Cancer Institute). Statistical significance was prespecified at *P* < 0.05 for findings in the entire study population.

**Figure 1:**
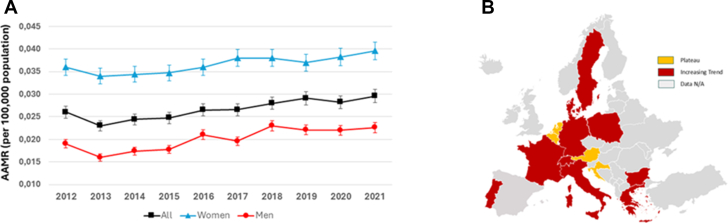
**Mitral Valve Prolapse-Attributable Mortality Rates and Trends** (A) Trends in age-adjusted mortality rates attributable to mitral valve prolapse in European countries, stratified by sex, from 2012 to 2020. No joinpoints were identified. (B) Map of Europe showing the mitral valve prolapse-attributable mortality trends. AAMR = age-adjusted mortality rate; N/A = data not available.

From 2012 to 2021, across the 12 European countries, a total of 25,240,710 deaths occurred. Among them, 1,634 deaths (1,103 men and 531 women) were attributed to MVP, corresponding to an overall mortality rate of 0.06 per 1,000 person-years or 6.44 deaths per 100,000 over the study period (proportional mortality of 0.006 deaths per 100 deaths from all causes). MVP-attributable mortality increased with age and was more frequently observed in women (71.8% of cases in patients aged ≥65 years of age) (Figure 1, Panel A). The AAMR for MVP-attributable mortality increased from 0.026 (95% CI: 0.023-0.029) per 100,000 population in 2012 to 0.030 (95% CI: 0.028-0.032) per 100,000 population in 2021 (AAPC: +2.3%; 95% CI: 0.6 to 4.0; *P* = 0.008). Men experienced a greater absolute increase in MVP-attributable mortality rates compared to women (p for parallelism 0.006). More precisely, in men, the AAMR increased from 0.019 (95% CI: 0.017-0.021) per 100,000 population in 2012 to 0.023 (95% CI: 0.021-0.024) per 100,000 population in 2021 (AAPC: +3.5% [95% CI: 0.4-6.6]; *P* = 0.03). In women, the AAMR increased from 0.036 (95% CI: 0.034-0.038) per 100,000 population in 2012 to 0.040 (95% CI: 0.037-0.042) per 100,000 population in 2021 (AAPC: +1.4% [95% CI: 0.4-2.4]; *P* = 0.008). The lowest AAMRs for MVP-attributed deaths were observed in Austria, the Netherlands, Croatia, and Denmark, while the highest was recorded in Italy. Notably, none of the countries analyzed showed a decline in MVP-attributed mortality rates. Conversely, the largest increases were observed in Italy (AAPC: +6.7%; 95% CI: 4.5-8.9; *P* < 0.001), followed by Bulgaria (AAPC: +4.6%; 95% CI: 3.0-6.2; *P* < 0.001), Denmark (AAPC:+4.3%; 95% CI: 2.2-6.5; *P* < 0.001), and Portugal (AAPC +4.1%; 95% CI: 2.1-5.6; *P* < 0.001) (Figure 1, Panel B). Because no joinpoints were identified, we additionally fitted a simple linear regression model to confirm the observed trends, which yielded comparable estimates of AAPC. Residual diagnostics, including the Durbin–Watson test (statistic range: 1.8-2.1), indicated no significant first-order autocorrelation, supporting the adequacy of a linear trend model.

## Discussion

This study provides an updated analysis of MVP-attributable mortality across Europe, highlighting a 98.1% increase over the past decade, particularly among older adults and women. The AAMR for MVP rose significantly from 2012 to 2021, with a pronounced surge in Italy.

The rising mortality trend is likely multifactorial. MVP prevalence increases with age and often coexists with cardiovascular conditions such as atrial fibrillation and heart failure, compounding mortality risk.^2^ Additionally, the growing burden of cardiometabolic risk factors, including hypertension, obesity, and diabetes, may have contributed to worse outcomes. Furthermore, advances in cardiovascular imaging, including echocardiography and cardiac magnetic resonance imaging, have improved MVP detection, potentially increasing its attribution in mortality reports. Moreover, enhanced medical coding and physician awareness and the recognition of malignant MVP, associated with SCD, have likely contributed to the observed increase.

Only 12 European countries reported MVP as an underlying cause of death during the study period. Many other countries either reported zero cases or did not classify MVP as the principal cause of death, which likely reflects differences in physician awareness, diagnostic emphasis, and national ICD coding practices. Therefore, present findings may not fully capture the diversity of MVP-attributable mortality across Europe, and caution is warranted when generalizing results to countries not included in the WHO data set, where epidemiological patterns and reporting practices may differ significantly. Moreover, historical factors, such as heightened concern over MVP-attributable SCD in the 1980s–1990s, may have influenced the propensity to attribute deaths to MVP in certain settings. Furthermore, the WHO mortality database relies on physician-completed death certificates, which are then coded by national statistics offices, rather than automated systems, and this process may vary substantially between countries. These factors may contribute to the observed geographic variability and limit the generalizability of our findings across Europe.

Despite these insights, study limitations should be considered. The reliance on ICD-10 coding introduces potential misclassification, and the WHO mortality data set reports only the primary cause of death, restricting the assessment of SCD in MVP cases.^4^ However, previous studies using the same ICD-10 codes as this analysis have demonstrated reasonable accuracy, with sensitivity for SCD ranging between 85% and 87%.^5^ The absence of autopsy confirmation may have resulted in overestimation or underestimation of MVP-attributable deaths. Additionally, differences in MVP diagnosis, management, and health care access across Europe may have contributed to variability in mortality rates. However, the use of the MVP code (I34.1), which specifically denotes nonrheumatic MVP, ensures that our data set accurately reflects MVP-attributable mortality independent of any confounding from rheumatic valve disease. Since the WHO mortality database relies on physician-certified and nationally coded causes of death, variations in certification and coding practices may have influenced MVP death reporting. Furthermore, because the database only records the underlying cause of death, secondary conditions such as severe mitral regurgitation, heart failure, and arrhythmias are not captured. The widespread diffusion of mitral transcatheter valve repair techniques could have positively affect our findings by potentially reducing the mortality rate linked with severe valvular heart disease. Finally, the lack of competitive cause-of-death information prevents reclassification of such cases and should be considered when interpreting the temporal trends we observed. All these limitations may have led to an underestimation of the broader mortality associated with MVP. Therefore, the present results must be cautiously interpreted and considered exploratory.

In conclusion, over the past decade, MVP-attributable mortality has risen across Europe, with variations based on demographics and geography. Further research is needed to better understand contributing factors and develop strategies to mitigate MVP-related mortality.

## Funding support and author disclosures

Dr Piazza has received consulting fees from BSC, Amgen, BCRI, PERC, NAMSA, BMS, Janssen, Regeneron; and has received research funding from 10.13039/100002491BMS/10.13039/100004319Pfizer, Janssen, 10.13039/100006396Alexion, 10.13039/100004326Bayer, 10.13039/100002429Amgen, 10.13039/100008497BSC, 10.13039/501100022336Esperion, and 10.13039/100000002NIH (1R01HL164717-01). Dr Boriani has received small speaker fees from Bayer, Boehringer Ingelheim, Boston, Daiichi Sankyo, Janssen, and Sanofi, outside of the submitted work; and is the principal investigator of the ARISTOTELES project (Applying ARtificial Intelligence to define clinical trajectorieS for personalized predicTiOn and early deTEction of comorbidity and muLtimorbidity pattErnS) that received funding from the European Union within the Horizon 2020 research and innovation programme (grant no. 101080189). All other authors have reported that they have no relationships relevant to the contents of this paper to disclose.
